# High-quality chromosome-level genome assembly of *Nicotiana benthamiana*

**DOI:** 10.1038/s41597-024-03232-0

**Published:** 2024-04-16

**Authors:** Seo-Rin Ko, Sanghee Lee, Hyunjin Koo, Hyojeong Seo, Jaewoong Yu, Yong-Min Kim, Suk-Yoon Kwon, Ah-Young Shin

**Affiliations:** 1 Plant Systems Engineering Research Center, Korea Research Institute of Bioscience and Biotechnology (KRIBB), Daejeon, 34141 Republic of Korea; 2 Department of Bioinformatics, KRIBB School of Bioscience, Korea University of Science and Technology (UST), Daejeon, 34113 Republic of Korea; 3 Biosystems and Bioengineering Program, KRIBB School of Biotechnology, Korea University of Science and Technology (UST), Daejeon, 34113 Korea; 4 UNGENE, Seoul, 08592 Republic of Korea; 5 Digital Biotech Innovation Center, Korea Research Institute of Bioscience and Biotechnology (KRIBB), Daejeon, 34141 Republic of Korea

**Keywords:** DNA sequencing, Polyploidy in plants

## Abstract

*Nicotiana benthamiana* is a fundamental model organism in plant research. Recent advancements in genomic sequencing have revealed significant intraspecific genetic variations. This study addresses the pressing need for a precise genome sequence specific to its geographic origin by presenting a comprehensive genome assembly of the *N. benthamiana* LAB strain from the Republic of Korea (NbKLAB). We compare this assembly with the widely used NbLAB360 strain, shedding light on essential genomic differences between them. The outcome is a high-quality, chromosome-level genome assembly comprising 19 chromosomes, spanning 2,762 Mb, with an N50 of 142.6 Mb. Comparative analyses revealed notable variations, including 46,215 protein-coding genes, with an impressive 99.5% BUSCO completeness score. Furthermore, the NbKLAB assembly substantially improved the QV from 33% for NbLAB360 to 49%. This refined chromosomal genome assembly for *N. benthamiana*, in conjunction with comparative insights, provides a valuable resource for genomics research and molecular biology. This accomplishment forms a strong foundation for in-depth exploration into the intricacies of plant genetics and genomics, improved precision, and a comparative framework.

## Background & Summary

*Nicotiana benthamiana* is an indispensable model organism in plant science, particularly for studying plant-microbe interactions and plant pathology due to its high susceptibility to various diseases, especially viral infections^1^. Its susceptibility to *Agrobacterium* has led to the use of agro-infiltration techniques for transient gene expression in leaf tissues^2^. In recent years, plant-derived systems, with *N. benthamiana* at the forefront, have become leading platforms for producing recombinant proteins, enzymes, vaccine antigens, antimicrobial peptides, diagnostic/research reagents, and monoclonal antibodies^3–6^. *N. benthamiana* plays a pivotal role in fundamental discoveries related to RNA interference, plant-pathogen interactions, metabolic pathway engineering, functional genomics, synthetic biology, and gene editing^7^. Despite its potential for biomanufacturing, challenges in achieving optimal yield and purity of protein products, often due to unintended protein degradation, persist^8^. *N. benthamiana*, belonging to the *Suaveolentes* section of the *Nicotiana* genus, is an allopolyploid believed to have originated from a single crossbreeding event. It possesses a basal haploid chromosome number of n = 12. Initially thought to have 24 chromosome pairs, subsequent polyploidization and chromosomal rearrangements have reduced the count of chromosomes to 19, resulting in a deficit of five chromosomes compared to the presumed ancestral state^9–12^. Short-read sequencing initially yielded fragmented drafts of the *N. benthamiana* genome^13,14^.

Recognizing the limitations of short-read sequencing, efforts have sought to explore long-read sequencing techniques. Two recent publications revealed a novel *N. benthamiana* draft genome using long-read sequencing. Kurotani *et al*. reported the genome using a PacBio Sequel II with seven SMRT cells^15^. In another paper, a hybrid approach that combined PacBio and Oxford Nanopore Technologies (ONT) sequencing platforms led to the creation of a high-quality genome assembly for the *N. benthamiana* LAB strain NbLAB360^16^. Comparative analyses highlighted disparities in single nucleotide polymorphism frequencies between NbLAB360 from the USA and EU laboratory accessions, emphasizing intraspecific genomic variations linked to geographical origin^16^. Additionally, we also analysed differences with the most recently published *N. benthamiana* genome Niben261^17^. A similar observation of breed-specific genomic variations across regions was also reported in a recent study of Korean native cattle^18^. These findings underscore the need for an accurate and high-quality genome sequence of the *N. benthamiana* LAB strain widely utilized in the Republic of Korea, NbKLAB.

In this investigation, we assembled a high-quality genome of *N. benthamiana* by using a combination of Illumina short reads, ONT long reads, and high-throughput chromosome conformation capture (Hi-C) data. This comprehensive approach yielded a genome assembly spanning 2,762 Mb, characterized by an N50 value of 142.6 Mb. Employing Hi-C scaffolding, we validated the presence of 19 chromosomes by utilizing the genome contact map. Furthermore, our efforts culminated in the identification of a total of 46,215 protein-coding genes, leading to an exceptional Benchmarking Universal Single-Copy Orthologs (BUSCO) score of 99.5%. This high-quality chromosomal-level genome assembly of NbKLAB establishes a robust cornerstone for prospective fundamental and applied research endeavors centered around *N. benthamiana*.

## Methods

### DNA extraction and genome sequencing

*N. benthamiana* Republic of Korea LAB (NbKLAB) plants were grown in standard fertilized soil under controlled environmental conditions at a constant temperature of 25°C with a 16-h light and 8-h dark photoperiod. 10 g of young leaves were collected from plants for 4 weeks, and high-molecular-weight genomic DNA was extracted. Nuclei were initially extracted from *N. benthamiana* cells using an *N. benthamiana* Nuclei Isolation Buffer (NIBM) (10 mM Tris-HCl pH 8.0, 10 mM EDTA pH 8.0, 100 mM KCL, 0.5 M sucrose, 4 mM spermidine, 1 mM spermine, and 0.15% β-mercaptoethanol). High-quality genomic DNA (gDNA) was obtained from these intact nuclei using a lysis buffer (50 mM Tris-HCl pH 7.5, 1.4 M NaCl, 20 mM EDTA pH 8.0, and 0.5% SDS). The quality of the isolated gDNA was assessed by measuring A_260_/_280_ absorbance ratios, which ranged from 1.8 to 2.0, using a Nanodrop 2000 spectrophotometer (Thermo Fisher Scientific, Waltham, MA, USA). To evaluate the concentration and purity of the gDNA, gel electrophoresis was performed. The size distribution of the gDNA fragments was determined using a TapeStation system (Agilent, Australia). Most gDNA fragments were distributed between 10 and 100 kb. The sequencing of *N. benthamiana* was conducted on three ONT PromethION R10.4 flow cell (FLO-PRO 112). Sequencing libraries were prepared according to the recommended protocols provided by ONT.

### Hi-C library preparation

Hi-C technology was also employed for chromosome-level genome assembly. Hi-C library construction protocol is as follows. Flower, root, and leaf tissue was mixed with 1% formaldehyde for fixing chromatin, and then the nuclei were isolated following a nuclei isolation method^19^. Fixed chromatin was digested with HindIII-HF (New England BioLabs), and we filled the 5′ overhangs with nucleotides and biotin-14-dCTP (Invitrogen) and ligated free blunt ends. After ligation, we purified DNA and removed biotin from unligated DNA ends. Fragmentation and size selection were performed to shear the Hi-C DNA. Hi-C library preparation was performed using the ThruPLEX® DNA-seq Kit (Takara Bio USA, Inc. Mountain View, CA, USA). The Hi-C library was evaluated by the distribution of fragment sizes with TapeStation D1000 (Agilent Technologies, Santa Clara, CA, USA) and sequenced in Illumina NovaSeq. 6000 (Illumina) with a length of 150-bp paired-end reads. To carry out Hi-C scaffolding analysis, 42.6 Gb (~15.3X) of NovaSeq data was generated.

### Genome *de novo* assembly

**Fig. 1:**
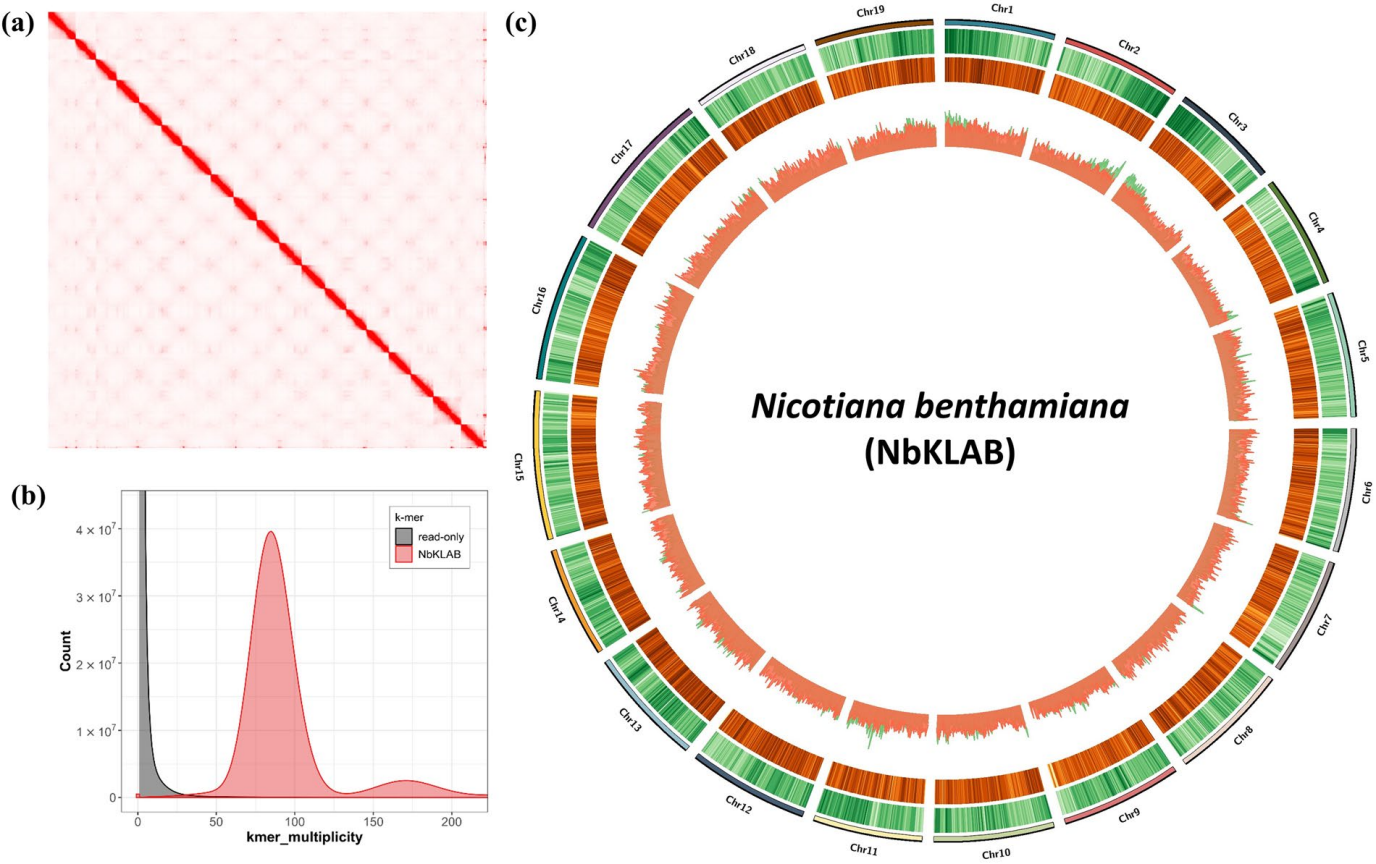
Genome assembly overview for NbKLAB. (**a**) Hi-C interaction map of NbKLAB. The heatmap illustrates the density of Hi-C interactions among distinct chromosomes. (**b**) Merqury assembly spectrum plots for evaluating k-mer completeness. (**c**) Circos plot displaying the NbKLAB genome assembly. Moving from the outer to inner circle, it shows gene density across chromosomes, the density of long terminal repeat retrotransposons (LTR-RTs), and the correlation between gene density and LTR-RT density.

To achieve a high-quality assembly, we initiated the process with rigorous quality control of the initial raw reads. Reads with a quality score below 7 and a length shorter than 5,000 bp were filtered out. Additionally, to remove chloroplast and mitochondria sequences, we obtained sequences from closely related species and conducted BLAST analysis. Subsequently, sequences with a query coverage of 80 or higher were removed, and Hi-C scaffolding was performed. As a result of these procedures, we endeavored to thoroughly eliminate potential contamination from chloroplast or mitochondrial genomes. Bascalling using Guppy v.6.1.1^20^ was carried out to eliminate low-quality reads, followed by read quality assessment using Nanoplot v.1.39^21^. A subsequent quality assessment conducted using Nanoplot v.1.39 provided insights into both the length and quality distributions of the reads. This led to the retention of 5,442,228 reads, spanning a total of 144,579,996 kb, with an N50 read length of 36,409. Next, we utilized NextDenovo v.2.5.0 (https://github.com/Nextomics/NextDenovo) to assemble the *N. benthamiana* genome using only the Nanopore long reads. The draft assembly was polished using NextPolish v.1.4.0^22^, first with long-read sequences used in the *de novo* assembly for one round, and then with short-read genome sequences produced by the Illumina sequencing platform for two rounds. Then we employed the Hi-C technology to obtain chromosome-level genome assembly. Firstly the paired-end Illumina reads were mapped onto the polished assembly using HiC-Pro v.3.1.0^23^ with default parameters to check the quality of the raw Hi-C reads. We obtained reads with approximately 15.3-fold coverage through Hi-C, with a total of 34,262,399 contacts, accounting for 25.37% of the filtered reads. Then Juicer v.2.13.07^24^ and 3D-DNA v.201008^25^ were applied to cluster the genomic contig sequences into potential chromosomal groups. Afterward, contig orientations were validated and ambiguous fragments were removed with manual curation using Juicebox v.1.11.08^26^, whereby consecutive contigs were linked to generate a high-quality genome assembly. The density of Hi-C interactions between chromosomes was confirmed through heatmap analysis and Hi-C matrix (Fig. 1a, Table 1). Our evaluation of k-mer completeness indicates that *N. benthamiana* possesses a paleopolyploid genome (Fig. 1b).

**Table 1:** Hi-C library statistics for NbKLAB genome.

	Long read + short read polishing	Long read + short read polishing + Hi-C scaffolding	
	Including scaffold	Including scaffold	Only chromosome
**Contigs**	140	668	19
**Total length**	2,792,201,312	2,792,183,363	2,762,242,804
**N50**	55,310,590	142,645,986	142,645,986
**Minimum length**	23,786	1,000	129,057,907
**Maximum length**	144,363,079	182,285,862	182,285,862
**GC**	37.84%	37.84%	37.75%
**BUSCO**	99.70%	99.60%	99.50%

We conducted a comparative analysis of the NbKLAB genome, NbLAB360, and Niben261 dataset to assess genome similarity. All three datasets featured the same 19 chromosomes. Quantitative metrics, including genome size (2,762,242,804 bp), maximum contig size (182,285,862 bp), and N50 values (142.6 Mb), revealed remarkable similarities the NbLAB360 dataset. However, the BUSCO v.5.3.2^27^ value for NbKLAB reached 99.5%, indicating a slightly superior assembly quality compared to the BUSCO values of NbLAB360 and Niben261, which are 98.5% and 98.7%, respectively (Table 2). Additionally, the Long Terminal Repeat (LTR) Assembly Index (LAI), a method for assessing genome assembly completeness by examining the accuracy of repeat sequence assemblies, was applied using LTR_retriever^28^. Additionally, we employed Circos v.0.69–9 software^29^ to depict the genome density features shown in Fig. 1c.

**Table 2:** Genome assembly and annotation statistics for NbKLAB and NbLAB360, and Niben261 genomes.

Species	NbKLAB	NbLAB360	Niben261
**Number of chromosome**	19	19	19
**N50 (Mb)**	142.6	143.1	152.6
**L50**	9	9	9
**Maximum (bp)**	182,285,862	182,027,195	194,605,305
**Genome size (bp)**	2,762,242,804	2,770,503,033	2,939,860,383
**GC (%)**	37.75	37.75	37.94
**BUSCO (%)**	99.5	98.5	98.7
**Protein-coding genes**	46,215	45,796	60,260

BUSCO: Benchmarking Universal Single-Copy Orthologs.

### Genome annotation

The annotation of protein-coding genes was conducted using the BRAKER2 sortware^30^. To obtain transcriptome data, RNA-seq reads^31^ were aligned to the NbKLAB reference genome using HISAT2 v.2.2.1^32^. Subsequent analysis utilized a protein database containing sequences from previously published, which were aligned to our genome assembly with ProtHint v.2.6.0^33^. Integration of these datasets was performed with GeneMark-ETP^33^ combining evidence from both transcriptomic and protein sequence alignments. The training and prediction of gene models were further refined using AUGUSTUS v.3.3.2^34^. The integration of predictions from AUGUSTUS and GeneMark-ETP was performed using TSEBRA^35^. To ensure the quality of predicted protein-coding genes, a filtration process was applied, utilizing BLASTP to remove sequences of poor quality based on specific criteria (E-value cut-off- 1e-10, Query coverage > 0.3, Subject Coverage > 0.3). Finally, we identified a total of 46,215 protein-coding genes.

### Comparative genomic analysis

**Fig. 2:**
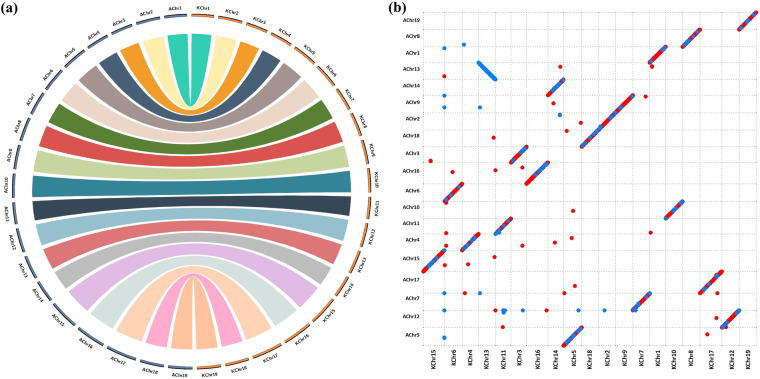
Comparative genomics. (**a**) Syntenic relationship between the NbKLAB and NbLAB360 genomes. (**b**) Comparison of all chromosomes of NbKLAB and NbLAB360 genomes using MUMmer plot. Alignment of whole genomes demonstrates a clear collinearity for all chromosomes. Dots distributed across the figure represent repetitive sequences aligning at various genomic locations. Red dots represent collinear sequences, while blue dots represent inverted sequences.

To compare genome sequences between NbKLAB and NbLAB360 at the chromosome level, we conducted pairwise comparisons using Circos v.0.69–9 and MUMmer4^36^. Protein sequences from both NbKLAB and NbLAB360 were aligned using BLASTP v.2.5.0. We identified conserved syntenic and collinearity blocks across the entire genome by employing the MCScanX program^37^. To focus on significant conserved genomic regions, we selected scaffolds larger than 1 Mb in length from all genomes for comparison. The results were then visualized using the Circos program (Fig. 2a). Additionally, we conducted sequence comparisons between chromosomes using Nucmer within the MUMmer4 software, with the parameters set as “-l 100, -c 500”. The MUMmer analysis revealed successful alignment of all 19 chromosomes between NbKLAB and NbLAB360 (Fig. 2b). These results demonstrate the accuracy of the alignment and establish comprehensive and accurate concordance within the genomic region.

### Repeat annotation

We employed an integrative approach that combined homology alignment and *de novo* prediction for repeat annotation. A repeat library was constructed from the assembled genomes using Utilizing RepeatModeler v.2.0.3^38^. Subsequent repeat annotation was conducted with RepeatMasker v.4.1.3^39^ (https://www.repeatmasker.org/). Comparatively, NbKLAB displayed a slightly higher detection of LTR elements at 1.40 Gb, constituting 49.99% of its entire genome, while NbLAB360 exhibited 1.37 Gb of LTR elements, accounting for 48.24% of its genome. In contrast, the distribution of SINE and LINE elements in NbKLAB was relatively reduced (Table 3).

**Table 3:** Comparative statistics of repetitive sequences in NbKLAB,NbLAB360, and Niben261 genomes.

	NbKLAB		NbLAB360		Niben261	
	Repeat length (bp)	Proportion (%)	Repeat length (bp)	Proportion (%)	Repeat length (bp)	Proportion (%)
**SINES**	1,142,314	0.04	3,259,793	0.11	937,157	0.03
**LINES**	105,513,178	3.78	115,717,888	4.08	118,975,624	3.92
**LTR elements**	1,395,831,879	49.99	1,367,691,325	48.24	1,002,900,094	33.04
**DNA transposons**	69,392,040	2.49	70,452,776	2.49	72,036,154	2.37
**Unclassified**	678,817,584	24.31	686,309,469	24.21	958,766,645	31.58

SINES: Short Interspersed Elements; LINES: Long Interspersed Elements; LTR: Long Terminal Repeat.

## Data Records

The raw sequencing data (Illumina, Nanopore, and Hi-C) used for genome assembly have been deposited in the NCBI Sequence Read Archive under the accession number PRJNA1034276^40^. The final genome assembly sequence of *N. benthamiana* cv. NbKLAB is available through the NCBI GenBank under accession number JAXGFW000000000^41^. Gene annotation data for *N. benthamiana* cv. NbKLAB has been submitted to the online open-access repository Figshare database^31^.

## Technical Validation

**Fig. 3:**
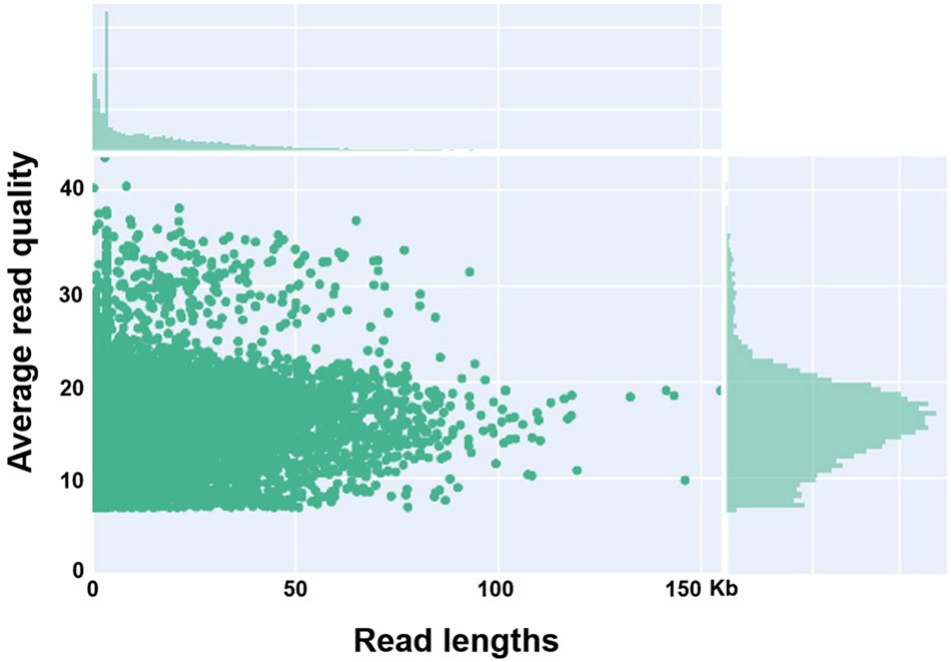
Raw data validation. Raw read length proportion and read quality.

We conducted a comprehensive evaluation of the quality and completeness of the raw ONT reads, totaling 9,000,040 reads. To assess the integrity of the raw reads, we employed Guppy v.6.1.1 to extract duplex bases and unpaired-simplex bases. The quality of the raw reads was analyzed using Nanoplot v.1.39(Fig. 3).

In this study, we employed a dual-reader approach with the ONT v10.3 platforms for genome sequencing, resulting in an impressive N50 read length of 34,701. The utilization of substantial-sized reads proved pivotal in enhancing the accuracy of our assembly process. This technological advancement substantially contributed to a more precise and comprehensive reconstruction of the genomic landscape compared to earlier *N. benthamiana* genome assemblies. The extended read sizes, made possible by the dual-reader strategy, underscore a significant enhancement in achieving a more robust and reliable genomic assembly, surpassing the earlier version.

**Fig. 4:**
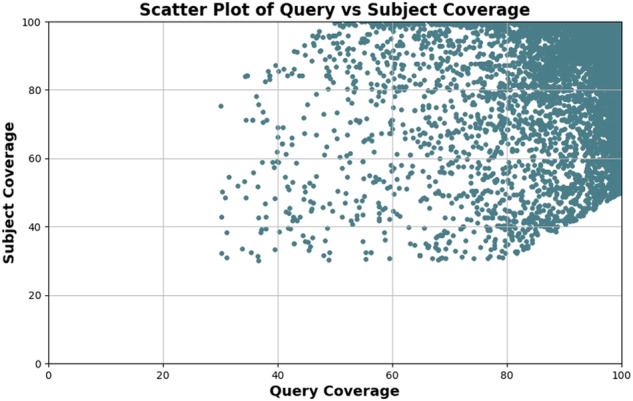
Quality Assessment of NbKLAB and NbLAB360 genes through BLASTP Analysis.

To assess the genome assembly completeness of NbKLAB and compare it to NbLAB360 and Niben261, we conducted a two-step validation. Firstly, we used paired-end illumina short reads to estimate the k-mer completeness score and the QV using Merqury v.1.3^42^. While NbLAB360 and Niben261 exhibited a commendable completeness score of 97.8% and 98.8%, respectively, reflecting solid genomic representation, NbKLAB surpassed this with an exceptional score of 99.4%. Furthermore, quality assessment revealed that NbLAB360 and Niben261 achieved a QV scores of 33 and 29.5, respectively, demonstrating the accuracy of the genome assembly. NbKLAB showcased a remarkable QV of 49, emphasizing its notable advancement and accuracy in genome reconstruction. Secondly, we predicted BUSCO completeness using a set of 1440 embryophyta genes^43^. Our analysis revealed that the NbKLAB genome assembly identified 99.5% of the conserved complete genes, whereas the NbLAB360 and Niben261 recognized 98.5% and 98.7%, respectively (Table 4). We utilized the BRAKER2 software for annotation and subsequently conducted BLASTP analysis using NbLAB360 to validate the annotation. The results revealed the identification of a total of 39,525 genes, with query coverage exceeding 90 and subject coverage surpassing 80, indicating a notably high-quality selection (Fig. 4). This suggests that the annotation process, validated through BLASTP analysis, has been effectively carried out. Collectively, these metrics emphasize the advancements achieved by our sequencing of NbKLAB, demonstrating significant improvements in assembly and annotation quality.

**Table 4:** Genome assembly validation.

	NbKLAB	NbLAB360	Niben261
**QV value**	49	31.5	29.5
**Merqury k-mer completeness score (%)**	99.4	97.8	98.8
**Scaffold N50 (Mb)**	142.6	145	151
**Complete BUSCOs (C) (%)**	99.5	98.1	98.7
**Complete and single-copy BUSCOs (S) (%)**	34.3	46	33.7
**Complete and duplicated BUSCOs (D) (%)**	65.2	52.1	65
**LTR Assembly index**	15.82	17.4	8.78

BUSCO: Benchmarking Universal Single-Copy Orthologs; LTR: Long Terminal Repeat; QV: Quality Value.

## Data Availability

All software employed for data processing was executed following the guidelines of the bioinformatic software cited above. If no detailed parameters are mentioned, the default parameters were used.
